# Superparamagnetic Iron Oxide Nanoparticle-Mediated Forces Enhance the Migration of Schwann Cells Across the Astrocyte-Schwann Cell Boundary *In vitro*

**DOI:** 10.3389/fncel.2017.00083

**Published:** 2017-03-28

**Authors:** Liangliang Huang, Bing Xia, Zhongyang Liu, Quanliang Cao, Jinghui Huang, Zhuojing Luo

**Affiliations:** ^1^Department of Orthopaedics, Xijing Hospital, The Fourth Military Medical UniversityXi'an, China; ^2^State Key Laboratory of Advanced Electromagnetic Engineering and Technology, Wuhan National High Magnetic Field Center, Huazhong University of Science and TechnologyWuhan, China

**Keywords:** spinal cord injury, magnetic nanoparticles, cell migration, cell transplantation, schwann cell, astrocyte

## Abstract

Schwann cells (SCs) are one of the most promising cellular candidates for the treatment of spinal cord injury. However, SCs show poor migratory ability within the astrocyte-rich central nervous system (CNS) environment and exhibit only limited integration with host astrocytes. Our strategy for improving the therapeutic potential of SCs was to magnetically drive SCs to migrate across the astrocyte-SC boundary to intermingle with astrocytes. SCs were firstly magnetized with poly-L-lysine-coated superparamagnetic iron oxide nanoparticles (SPIONs). Internalization of SPIONs showed no effect upon the migration of SCs in the absence of a magnetic field (MF). In contrast, magnetized SCs exhibited enhanced migration along the direction of force in the presence of a MF. An inverted coverslip assay showed that a greater number of magnetized SCs migrated longer distances onto astrocytic monolayers under the force of a MF compared to other test groups. More importantly, a confrontation assay demonstrated that magnetized SCs intermingled with astrocytes under an applied MF. Furthermore, inhibition of integrin activation reduced the migration of magnetized SCs within an astrocyte-rich environment under an applied MF. Thus, SPION-mediated forces could act as powerful stimulants to enhance the migration of SCs across the astrocyte-SC boundary, via integrin-mediated mechanotransduction, and could represent a vital way of improving the therapeutic potential of SCs for spinal cord injuries.

## Introduction

After spinal cord injury, cysts are always formed at the injury site both in humans and rats, which are inhibitory for nerve regeneration (Schwab, 2002). Therefore, cell transplantation therapy remains a highly attractive approach to fill these cysts and provide an environment suitable for regeneration. Thus far, a variety of cells have been introduced for SCI repair, including Schwann cells (SCs), oligodendrocytes, olfactory ensheathing cells (OECs), and stem cells. Of these cells, the SC is considered to be one of the most promising candidates for autologous transplantation, since SCs not only provide trophic and physical growth-permissive substrates for axonal regrowth, but also form myelin for functional recovery (Xu et al., 1997; Campos et al., 2004; Houle et al., 2006; Papastefanaki et al., 2007; Zujovic et al., 2012). Previous research has shown that the transplantation of SCs results in successful axonal regeneration into the grafts and partial functional recovery in both rodent and primate models of SCI (Takami et al., 2002; Pearse et al., 2004; Schaal et al., 2007; Guest et al., 2013). In addition, the harvesting and expansion of SCs has been well developed via *in vitro* systems, which presents us with a unique opportunity for future clinical applications (Rutkowski et al., 1995). However, SCs show limited migratory ability in an astrocyte-rich environment and are unable to integrate with host astrocytes, leading to the formation of a sharp boundary between the SC graft and the host tissue astrocytes (Franklin and Blakemore, 1993; Shields et al., 2000; Lakatos et al., 2003; Grimpe et al., 2005; Wiliams and Bunge, 2012). Therefore, regenerating axons can regenerate into the SC graft, but fail to depart from the bridging graft back into the distal host spinal cord; this represents a significant limitation in the efficacy of using SCs to repair SCI.

Successful regeneration of an injured central nervous system (CNS) requires that transplanted SCs penetrate the astrocyte-SC boundary and guide regenerating axons to reach their final destination. Thus, it is of great importance to enhance the migration of SCs in the astrocyte-rich CNS and increase the integration of SCs and astrocytes during the repair of SCI. Over past decades, various strategies have been proposed to enhance the migrating ability of SCs in an astrocyte-rich environment, including the over-expression of polysialylated neural cell adhesion molecule (PSA-NCAM; Papastefanaki et al., 2007; Luo et al., 2011; Ghosh et al., 2012), the knockdown of aggrecan or N-cadherin, and by blocking the EphA receptor (Fairless et al., 2005; Afshari et al., 2010). However, the enhanced migration of SCs in these studies appeared to be random, with no preferred migration direction, thus limiting the efficiency of SCs to penetrate across the astrocyte boundary and migrate into the distal spinal cord. Therefore, enhancing the migration of SCs in a controlled and desired direction would be highly beneficial for SCI repair.

Superparamagnetic iron oxide nanoparticles (SPIONs) have been widely used as magnetic resonance imaging (MRI) contrast agents. SPIONs show strong magnetization in the presence of a magnetic field (MF), and retain no permanent magnetization upon removal of the field (Liu et al., 2015). This unique feature of SPIONs has led to several successful applications, including biological separation, drug delivery and stem cell labeling (Yang et al., 2004; Zhang et al., 2009; Eamegdool et al., 2014). Transplanting cells loaded with SPIONs can be successfully delivered to the specific injury tissue using applied fields (Nishida et al., 2006; Song et al., 2010; Fujioka et al., 2012). In addition, a more recent study has confirmed that the orientation of the neuronal growth process can be directed via magnetic nanoparticles under an applied MF (Riggio et al., 2014). Thus, if SPIONs are incorporated into SCs, a strong magnet could exert force upon the intracellular SPIONs and thus direct the migration of SCs into the astrocyte-rich area and increase the intermingling of SCs and astrocytes. The present study was designed to investigate such a possibility.

When a physical force acts upon a cell, in addition to the direct effect of physical changes, the cell can also sense the physical force and convert it into a biochemical signal (a process known as mechanotransduction; Sun Z. et al., 2016; Poitelon et al., 2017). Integrin has been demonstrated to mediate mechanotransduction in various types of cells, including neurons, fibroblasts, epithelial cells, cardiomyocytes, and tendon stem/progenitor cells (Ye et al., 2010; Zhang et al., 2011; Moore et al., 2012; Fiore et al., 2015; Israeli-Rosenberg et al., 2015; Sun X. et al., 2016; Sun Z. et al., 2016; Wang et al., 2016). Recent studies have also shown that integrin plays a vital role in cell migration; the migration of SCs on astrocytes was shown to be clearly integrin-dependent (Nodari et al., 2007; Afshari et al., 2010). Activation of integrin results in the enhanced migration of SCs even in an astrocyte-rich environment. Thus, it is interesting to investigate whether integrin is activated and mediates the mechanotransduction mechanism involved in the magnetic force driving the migration of SCs into astrocytes.

In the present study, poly-L-lysine coated SPIONs (PLL-SPIONs) were firstly synthesized and characterized. The toxicity of PLL-SPIONs was then determined by PrestoBlue assay and live-dead assay. The magnetization of SCs and the cellular localization of SPIONs were identified by scanning electron microscopy (SEM) and transmission electron microscopy (TEM). The inverted coverslip assay and confrontation assay were also used to investigate the migration ability of magnetized SCs in the presence of astrocytes under a MF. Furthermore, we used an integrin antibody to inactivate integrin in order to investigate whether integrin mediates the mechanotransduction involved in the enhanced migratory ability of magnetized SCs in an astrocyte-rich environment under an applied MF.

## Materials and methods

### Preparation of PLL-SPIONs

SPIONs were synthesized using methodology described previously (Liu et al., 2015). In brief, 0.486 g NaOH (Sigma-Aldrich, USA) and 1.364 g KNO_3_ (Acros Organics, Belgium) were dissolved in 135 ml deionized water and bubbled with N_2_. Then, 15 mL of 0.01 M H_2_SO_4_ (Panreac, Spain) solution containing 0.308 g FeSO_4_·7H_2_O (Sigma-Aldrich) was added dropwise under constant stirring. After the precipitation was completed, N_2_ flow was allowed to pass for another 10 min. The suspension was kept at 90°C for 24 h. Finally, the product was cooled in an ice bath. The synthetic product was then separated via magnetic decantation, and washed three times with deionized water.

To coat the naked SPIONs with PLL, 10 mg SPIONs were resuspended in 0.1% PLL (≤150 kD; Sigma-Aldrich) solution and sonicated overnight. Then, the solution was washed with deionized water to remove uncoated PLL and was then stored in a refrigerator at 4°C.

### Characterization of PLL-SPIONs

The morphological characteristics and mean size of the PLL-SPIONs generated were evaluated under a transmission electron microscope (H-600, HITACHI, Tokyo, Japan). Magnetic measurements were carried out using a vibrating sample magnetometer (665; Lake Shore Cryotronics, USA). Zeta potential was analyzed at room temperature utilizing a zeta potential analyzer (Beckman Coulter, USA).

### Isolation and purification of SCs and astrocytes

This study was carried out according to the recommendations of the Guide for the Care and Use of Laboratory Animals (National Institutes of Health Publication No. 85-23, revised 1985). All experiments were performed following approval from the Institutional Ethical Committee of the Fourth Military Medical University.

SCs from postnatal day 2 (P2) newborn Sprague-Dawley (SD) rats were isolated and purified from sciatic nerves following our established protocol (Huang et al., 2015). The purity of SC cultures was determined by immunofluorescence for p75^*NTR*^ protein (Figures S1A–C); the purity of the SCs obtained was more than 95%.

Primary astrocytes were isolated from cerebral cortices of neonatal (P2) SD rats as described previously (Afshari et al., 2010). First, brains were removed and demembranated using a dissecting microscope. Then, tissues were chopped into 0.5 mm^3^ and digested with 0.1% trypsin for 30 min. The enzyme solution was then removed, and DMEM containing 10% fetal bovine serum (FBS) was added and titrated gently. The minced tissue was centrifuged, and resuspended in DMEM with 10% FBS, and plated on 75 cm^2^-flasks precoated with Poly-D-lysine (PDL). After 7–10 days, microglia and oligodendrocyte precursor cells were removed by shaking for 20 h at 200 rpm at 37°C. Next, the astrocytes were washed twice to remove floating cells and the medium was replaced with fresh medium. Purity of the astrocyte culture was determined by immunofluorescence for GFAP protein (Figures S1D–F); purity of the astrocytes obtained was more than 90%.

### Prestoblue assay

The PrestoBlue assay was used to study the cytotoxicity of PLL-SPIONs in accordance with the manufacturer's instructions (Life Technologies, USA). In brief, SCs were incubated with different concentrations of PLL-SPIONs (0–100 μg/ml) for 24 h, 48 h, and 72 h, respectively. Then, cells were washed with PBS, and a mixture of 90-μl fresh medium and 10-μl PrestoBlue reagent was introduced into the samples, followed by incubation at 37°C for 2 h. Thereafter, optical density was measured at 570/600 nm using a microplate reader. All assays were conducted in triplicate.

### Live-dead assays

To further investigate the potential toxicity of PLL-SPIONs upon SCs, a live-dead assay was conducted in accordance with the manufacturer's instructions (BioVison Inc., USA). Briefly, cells were incubated with different concentrations of PLL-SPIONs (0–100 μg/ml) for 24 h, 48 h, and 72 h, respectively. Then, SCs were washed, and the staining solution was introduced to each sample, followed by incubation at 37°C for 15 min. Cellular viability was then observed by fluorescence microscopy (DM6000; Leica, Germany); living cells were labeled green and dead cells were labeled red.

### Real-time polymerase chain reaction (RT-PCR)

Total RNA was isolated from control cells and cells incubated with 10 μg/ml PLL-SPIONs for 24 h. cDNA was then synthesized using Superscript III reagents according to the manufacturer's instructions (Invitrogen, USA). RT-PCR was then conducted using an Eppendorf Master Cycler EP Realplex Thermal Cycler and the iQ SYBR Green Supermix (Bio-Rad, USA) in accordance with the manufacturer's instructions. The primers for glial cell line-derived neurotrophic factor (GDNF), brain-derived neurotrophic factor (BDNF), nerve growth factor (NGF), neurotrophin-3 (NT-3) and β-Actin (internal control) are shown in Table 1. RT-PCR conditions were as follows: denaturation at 95°C for 30 s; primer annealing at 59°C for 30 s; and elongation at 72°C for 40 s. Quantification of PCR products was performed using the 2-ΔΔCt method. Quantities of mRNA were normalized to the housekeeping gene, β-Actin. All assays were performed three times using triplicate wells.

**Table 1:** **Primer sequences used for RT-PCR**.

**Gene**	**GenBank accession no**.	**Direction length**	**Sequence**	**Length (bp)**
GDNF	NM_019139.1	Upper	5′AGAGGGAAAGGTCGCAGAG 3′	142
		Lower	5′ CTTCACAGGAACCGCTACAA 3′	
BDNF	NM_001270630	Upper	5′ GCCCAACGAAGAAAACCATA 3′	98
		Lower	5′ CCAGCAGAAAGAGCAGAGGA 3′	
NGF	NM_001277055	Upper	5′CAGGCAGAACCGTACACAGA 3′	183
		Lower	5′ AAACAGTTTGGGGTCCACAG 3′	
NT-3	NM_031073.3	Upper	5′GATCCAGGCGGATATCTTGA 3′	162
		Lower	5′GCGTCTCTGTTGCCGTAGT 3′	
β-Actin	NM_031144.3	Upper	5′ CCCATCTATGAGGGTTACGC 3′	150
		Lower	5′ TTTAATGTCACGCACGATTTC 3′	

### SEM and TEM analysis of cellular localization of SPIONs

To analysis the presence of PLL-SPIONs on the cell membrane, SCs were grown on coverslips precoated with PDL and incubated with PLL-SPIONs (10 μg/ml). Twenty-four hours after incubation, SCs were washed with PBS, fixed and dehydrated with serial ethanol solutions. The samples were then dried under vacuum at room temperature, sputter-coated with gold, and examined under a scanning electron microscope (S-3400N, Hitachi, Japan).

To analysis the internalization of SPIONs into SCs, SCs were incubated with PLL-SPIONs (10 μg/ml) for 24 h. For TEM analysis, cells were washed with PBS, pelleted, fixed, and osmicated. Then, the specimens were dehydrated in ascending alcohols. After drying, specimens were embedded in a solution of TAAB resin (TAAB Laboratories, England, UK) and cut in 70 nm thin slices (Riggio et al., 2014). The ultrathin sections were examined under a transmission electron microscope (HITACHI, Japan).

### Quantification of intracellular SPIONs

The intracellular SPIONs were quantified by measuring the amount of iron in the cells according to a method described previously (Kim et al., 2011). Briefly, PLL-SPIONs were added to the cells at concentrations ranging from 0 to 100 μg/ml. Twenty-four hours after incubation, the cells were washed three times with ice cold PBS containing deferoxamine (1 mM), an iron chelator to remove the uninternalized PLL-SPIONs. Then, the cells were detached, counted, and lysed in 27.75% HCl to dissolve all the components including nanoparticles. The specimens were diluted (1:4) with DI water and filtered. The iron concentration was examined by an inductive coupled plasma-atomic emission spectrometer (ICP-AES, ICPS-7500, Shimadzu, Japan).

To investigate the amount of intracellular SPIONs overtime in culture, SCs were firstly magnetized with 10 μg/ml PLL-SPIONs for 24 h. Then, the cells were washed three times with PBS containing deferoxamine (1 mM) to remove the uninternalized PLL-SPIONs. This time point was defined as 1 d. Thereafter, the cells were incubated with fresh medium. At 1 d, 3 d, 5 d, and 7 d, the intracellular iron was measured as described above.

### Inverted coverslip migration assay

The inverted coverslip assay was carried out following previous description (Cao et al., 2007). Non-magnetized SCs or magnetized SCs were plated onto coverslip fragment (~5 × 5 mm^2^) precoated with PDL. The cells were allowed to attach for 24 h. Then, removed the loosely attached cells by washing with culture medium, and inverted the cells facing downward onto the PDL substrates or astrocytes. The applied MF (neodymium cubic magnet, N48, residual induction 1.4 T, cube side 50 mm) was placed parallel to one edge of the coverslip for 2 d, and marked the edge for further examination. The maximum distance of cells that away from the edge of the coverslip, and the number of cells migrating at each distance was measured.

### Confrontation assays

The confrontation assay was performed as described previously (Lakatos et al., 2000). Briefly, a 10-μl strip containing 10,000 SCs was set up opposing a parallel 10-μl strip containing 10,000 astrocytes. Non-attached cells were removed after 1 h. A magnet was then placed parallel to the strip of SCs. Cultures were then maintained in DMEM with 10% FBS, and allowed to grow toward each other over a period of 7 days, giving time for cells to make contact and interact. Cultures were then immunolabeled using anti-GFAP and anti-p75^NTR^. The number of cells which had successfully migrated across the cell-cell boundary into the astrocyte domain was then counted. Six areas were randomly chosen, and the boundaries covered an approximate distance of 300 μm. To assess the hypertrophy of astrocytes in contact with magnetized SCs or non-magnetized SCs, the mean area of GFAP immunoreactivity (GFAP-ir) in astrocytes was calculated from three separated experiments using ImageJ software 1.46 m (http://rsb.info.nih.gov/ij/).

To investigate whether the activation of integrin was involved in the enhanced migratory ability of magnetized SCs in an astrocyte-rich environment under MF, 10 μg/ml of beta-1 integrin blocking antibody (BD Horizon™, USA) was added to cell cultures after 1 h of attachment; cultures were then maintained and stained as described above.

### Immunocytochemistry

Cell cultures were fixed with 4% paraformaldehyde for 20 min, blocked with 0.25% Triton-X/10% normal goat serum for 1 h. Then, specimens were incubated with primary antibodies overnight at 4°C. The next day, specimens were rinsed four times in PBS, and incubated with appropriate fluorochrome-labeled secondary antibodies and DAPI (Abcam Inc., UK). The following primary antibodies were used: polyclonal chicken anti-glial fibrillary acidic protein (GFAP; 1:1000, Abcam Inc., UK) for astrocytes, and polyclonal rabbit anti-p75 (1:50, Abcam Inc., UK) for SCs.

### Statistical analysis

All values are presented as means ± standard deviation (*SD*). One-way analysis of variance (ANOVA) was used for the statistical comparison of means. Significant results were then assessed by Tukey's *post hoc* testing (GraphPad Prism 6.0). A difference of *p* < 0.05 was considered statistically significant.

## Results

### Characterization of PLL-SPIONs

**Figure 1:**
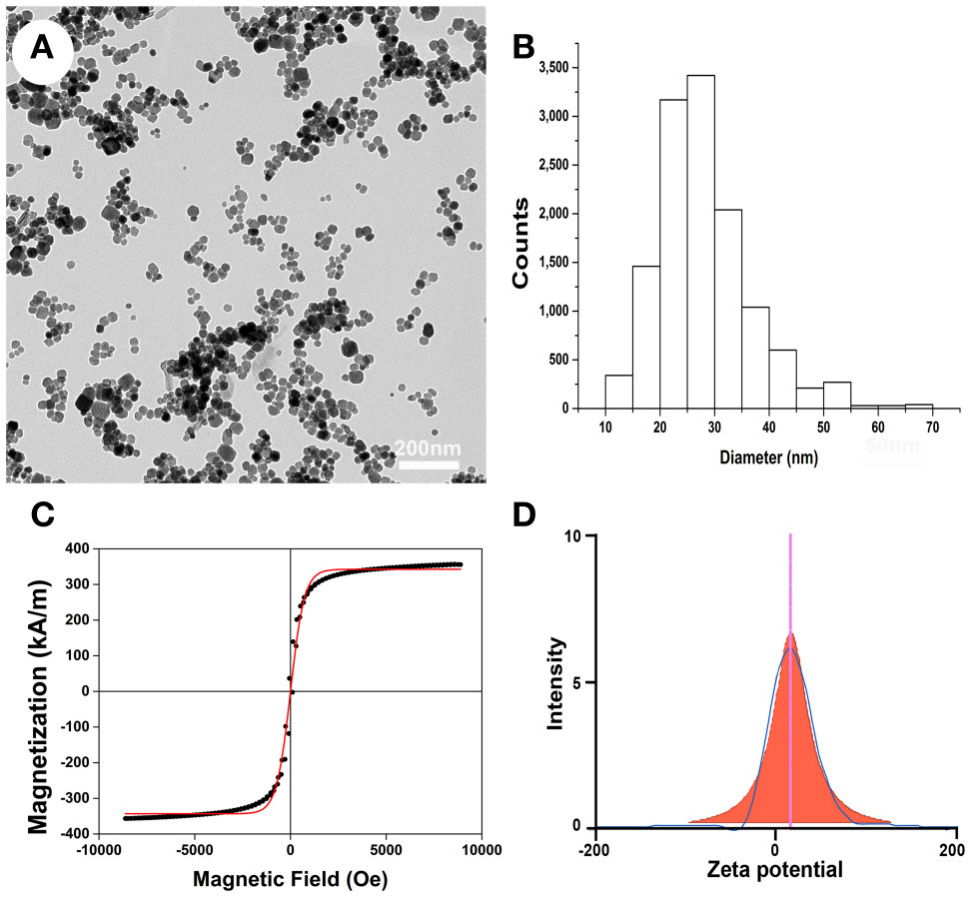
**Characterization of PLL-SPIONs. (A)** TEM image of PLL-SPIONs. **(B)** The overall distribution of particle size. **(C)** The magnetization curve of PLL-SPIONs at 298 k. **(D)** The zeta potential of PLL-SPIONs.

TEM was used to characterize the particle size and morphological features of the PLL-SPIONs. The magnetite (Fe_3_O_4_) core exhibited a mean diameter of 25 nm (Figures 1A–B) while the PLL-SPION particles showed uniformity in distribution and were mostly spherical in shape. The magnetization curve of the PLL-SPIONs showed a symmetrical hysteresis loop, which is characteristic of superparamagnetic nanoparticles. At room temperature, PLL-SPIONs showed no coercivity and remanence, further confirming the superparamagnetic characteristics of these nanoparticles. The saturation magnetization at 298 K was 351.6 kA/m (Figure 1C). The zeta potential of the PLL-SPIONs exhibited a positive charge (~ +15 mV) at physiological pH = 7.0 (Figure 1D), indicating successful functionalization of the SPIONs surface by PLL.

### The effect of PLL-SPIONs upon the function of SCs

**Figure 2:**
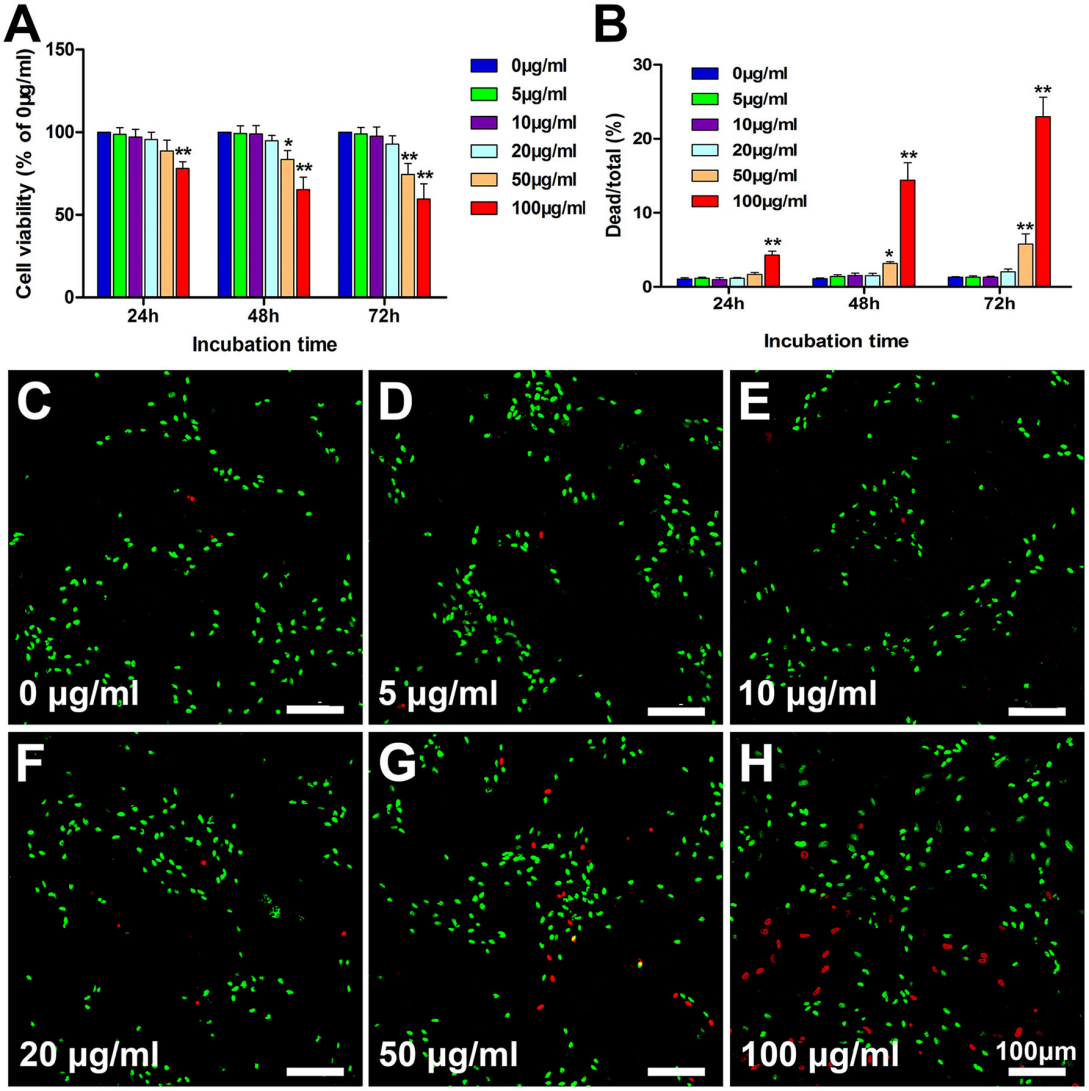
**Assessment of the cytotoxicity of PLL-SPIONs. (A)** Cell viability evaluated by the PrestoBlue assay. **(B)** Cell viability evaluated by the live-dead assay. **(C–H)** Representative images from the live-dead assay 72 h after co-incubation at all tested concentrations. Live cells were labeled green, while dead cells were labeled red. Scale bar = 100 μm. Data are expressed as means ± *SD*; ^*^p < 0.05, ^**^p < 0.01.

Following the co-incubation of PLL-SPIONs with SCs, the cytotoxicity of PLL-SPIONs was evaluated by a fast and sensitive live PrestoBlue assay at 24 h, 48 h, and 72 h. At 24 h after co-incubation, the nanoparticles exhibited no significant toxicity at concentrations ranging from 5 to 50 μg/ml (Figure 2A), and the percentage of dead cells ranged from 1 to 2% (Figure 2B). With the extension of incubation time, a significant reduction in cell viability was observed. At 72 h, the PrestoBlue assay showed a reduction of 74% at 50 μg/ml and 59% at 100 μg/ml, respectively (Figure 2A). The percentage of dead cells increased to 5.18 ± 1.38% (50 μg/ml) and 22.99 ± 2.63% (100 μg/ml), which was significantly higher than that of the unlabeled cells (Figures 2B–H). These findings suggest that the cytotoxicity of PLL-SPIONs was both time and dose dependent. PLL-SPIONs with a concentration 20 μg/ml showed no statistically significant cytotoxicity for SC magnetization at the observed time points, although cell viability showed a downward trend at 20 μg/ml. Thus, to avoid such potential cytotoxicity, we choose a concentration of 10 μg/ml, rather than the threshold concentration of 20 μg/ml, for the remainder of the experiments.

**Figure 3:**
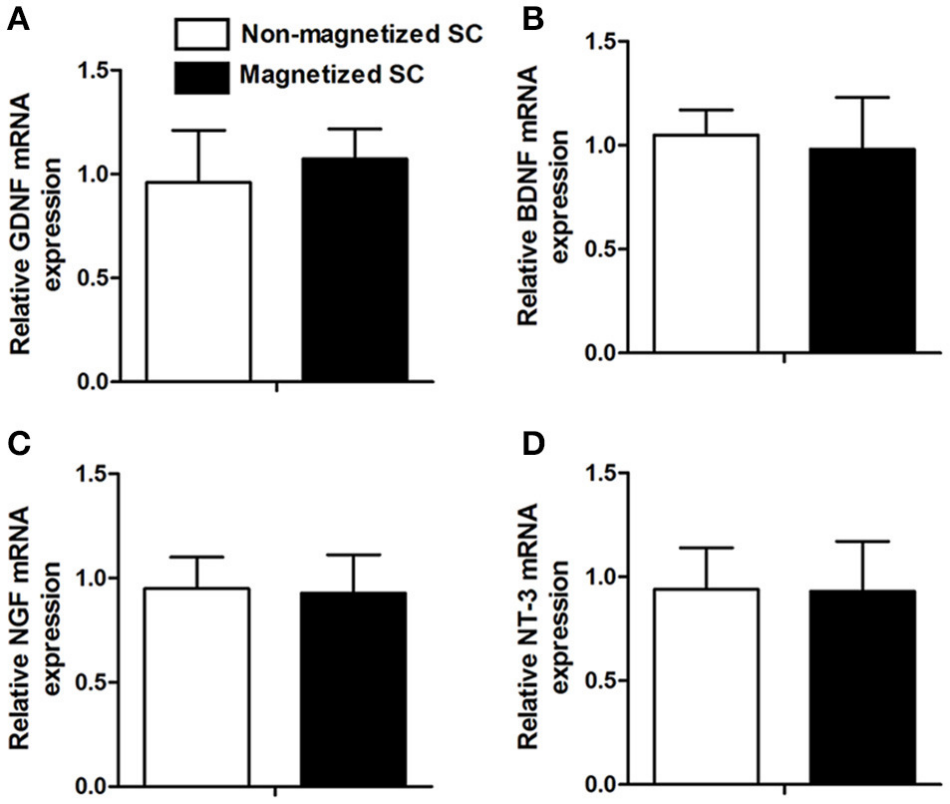
**mRNA levels of neurotrophic factors 24 h after the magnetization of SCs**. Relative mRNA expression of GDNF **(A)**, BDNF **(B)**, NGF **(C)** and NT-3 **(D)**.

In addition to cell viability, other functions, such as the expression of neurotrophic factor, are of great importance for further *in vivo* application. RT-PCR was conducted to investigate for differences in the expression of GDNF, BDNF, NGF, and NT-3 between control cells and cells incubated with 10 μg/ml of PLL-SPIONs for 24 h (Figure 3). Our results showed that the expression levels of these genes in magnetized SCs were similar to those in non-magnetized SCs, indicating that a proper concentration of PLL-SPIONs had negligible effect upon the function of SCs.

### Cellular localization and internalization of PLL-SPIONs

**Figure 4:**
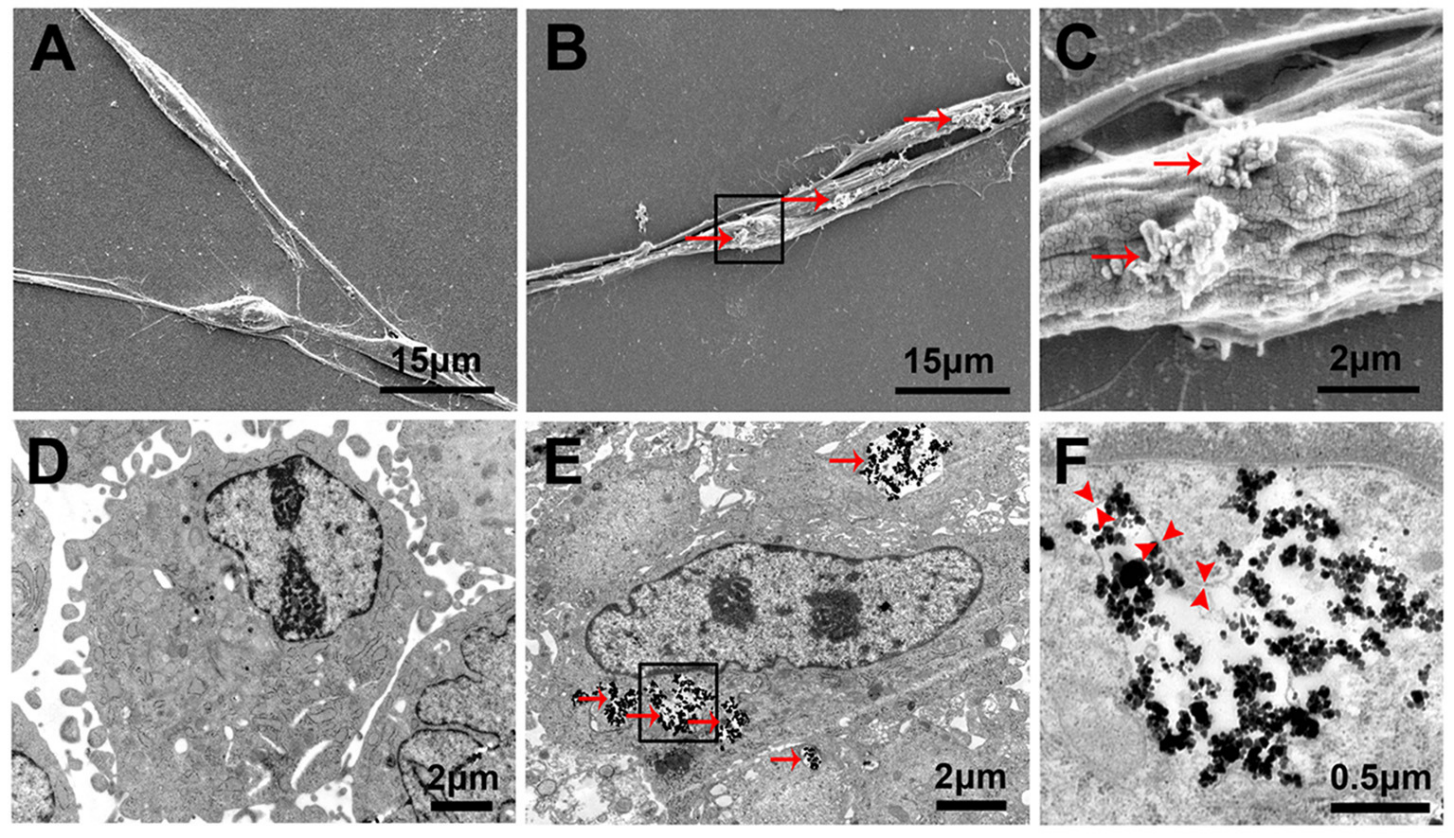
**Cellular localization and internalization of PLL-SPIONs**. SEM analysis of non-magnetized SCs **(A)** and SCs incubated with 10 μg/ml PLL-SPIONs for 24 h **(B,C)**. Red arrows show SPION agglomerates (bright spots) on the cell membrane. TEM image of non-magnetized SCs **(D)** and SCs incubated with 10 μg/ml PLL-SPIONs for 24 h **(E)**. SPIONs remained dispersed as single SPIONs and localized within intracellular vesicles. Red arrows: particles in the cytoplasm. **(F)** High magnification of the boxed area in **(E)**. Red arrow head: the membrane of endosomes.

Twenty-four hours after the incubation of SCs with PLL-SPIONs (10 μg/ml), SEM revealed that a few particles had been attached on the cell surface, which was not evident with the non-magnetized SC cells (Figures 4A–C). TEM images showed that SPIONs had been taken up by SCs. The internalized SPIONs were localized in endosomes within the cytoplasm of SCs (Figures 4D–F). In addition, the presence of intracellular SPIONs was also confirmed by the quantification of intracellular iron, which increased in line with incubation concentrations (Table 2). We further examined the amount of intracellular iron over culture time. The amount of intracellular iron showed a decreasing trend over time. After one day, the quantity of intracellular iron was 1.27 ± 0.08 pg/cell. With the extension of time, the amount of iron per cell decreased to 0.67 ± 0.05 pg/cell at 7 d, which was approximately half the concentration seen on day 1 of culture (Table 3).

**Table 2:** **Quantification of intracellular iron with different incubation concentrations**.

**Incubation concentrations (μg/ml)**	**5**	**10**	**20**	**50**	**100**
Quantification of intracellular iron (pg/cell)	0.48 ± 0.03	1.27 ± 0.08	1.58 ± 0.15	1.89 ± 0.16	2.23 ± 0.25

**Table 3:** **Quantification of intracellular iron over time during *in vitro* culture**.

**Time point (d)**	**1**	**3**	**5**	**7**
Quantification of intracellular iron (pg/cell)	1.27 ± 0.08	1.12 ± 0.11	0.83± 0.07	0.67 ± 0.05

### Directed and enhanced migration of magnetized SCs under an applied MF

**Figure 5:**
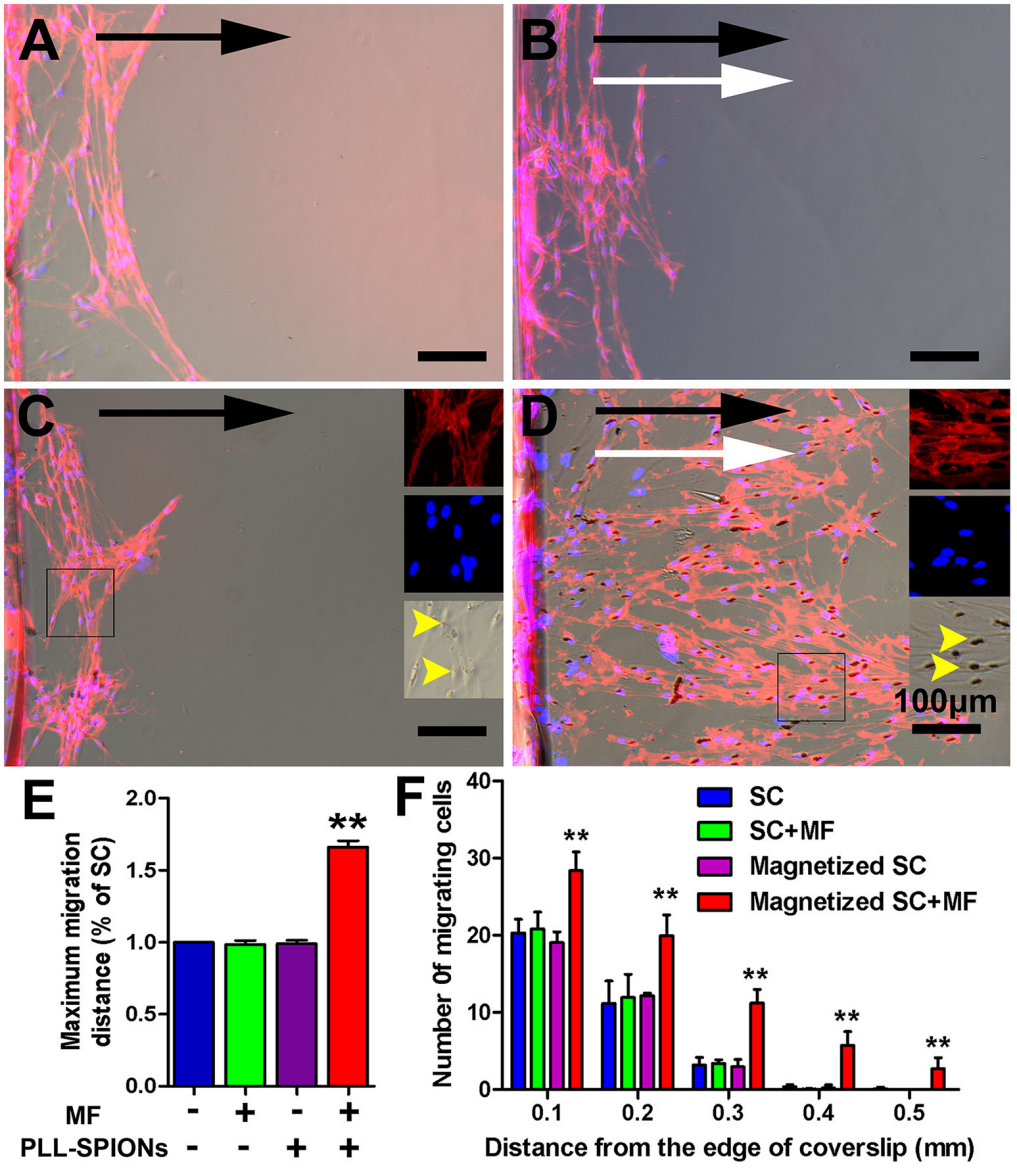
**Directed and enhanced migration of magnetized SCs under an applied MF. (A–D)** Representative images of SC migration for non-magnetized SCs without a MF (SCs), non-magnetized SCs with a MF (SCs + MF), magnetized SCs without MF (magnetized SCs) and magnetized SCs with MF (magnetized SCs + MF) group, respectively. Black arrows point in the direction of SC migration; MF was applied for 2 d while white arrows point in the direction of the MF; yellow arrow heads point to internalized SPIONs. **(E)** Mean maximum distance traveled from the edge of the inverted coverslip. **(F)** Number of cells migrated from the edge of the inverted coverslip. SCs were immunolabeled for P75 (red) while nuclei were labeled with DAPI (blue). Scale bar = 100 μm. Data are expressed as means ± *SD*; ^**^p < 0.01.

To evaluate the migration ability of magnetized SCs under an applied MF, the inverted coverslip assay was conducted, as shown in Figure 5. No significant difference was observed in the number of cells migrating from the edge of the coverslips among non-magnetized SCs with or without a MF, and magnetized SCs without MF (Figures 5A–C), indicating that intracellular SPIONs, or a MF alone, has no effect upon the migration of SCs. However, in the presence of a MF, the magnetized SCs actively migrated toward the region of maximum field density (Figure 5D). The number of magnetized SCs migrating from the edge of the coverslips was 1.93 times higher under an applied MF than that without field stimulation (^**^p < 0.01). Similarly, the mean maximum migration distance of SCs was longer in the magnetized SC with a MF than that in the other three groups (^**^p < 0.01). In addition, no preferable direction was found in the orientation of migration for non-magnetized SCs with or without a MF, and magnetized SCs without field stimulation. In contrast, magnetized SCs tended to be arranged in parallel to the magnetic force in the presence of a MF. Furthermore, SPIONs were dispersed in the cytoplasm of SCs (Figure 5C) but aggregated to the region of maximum field density in the presence of a MF (Figure 5D).

### Increased migration of magnetized SCs on astrocytes under an applied MF

**Figure 6:**
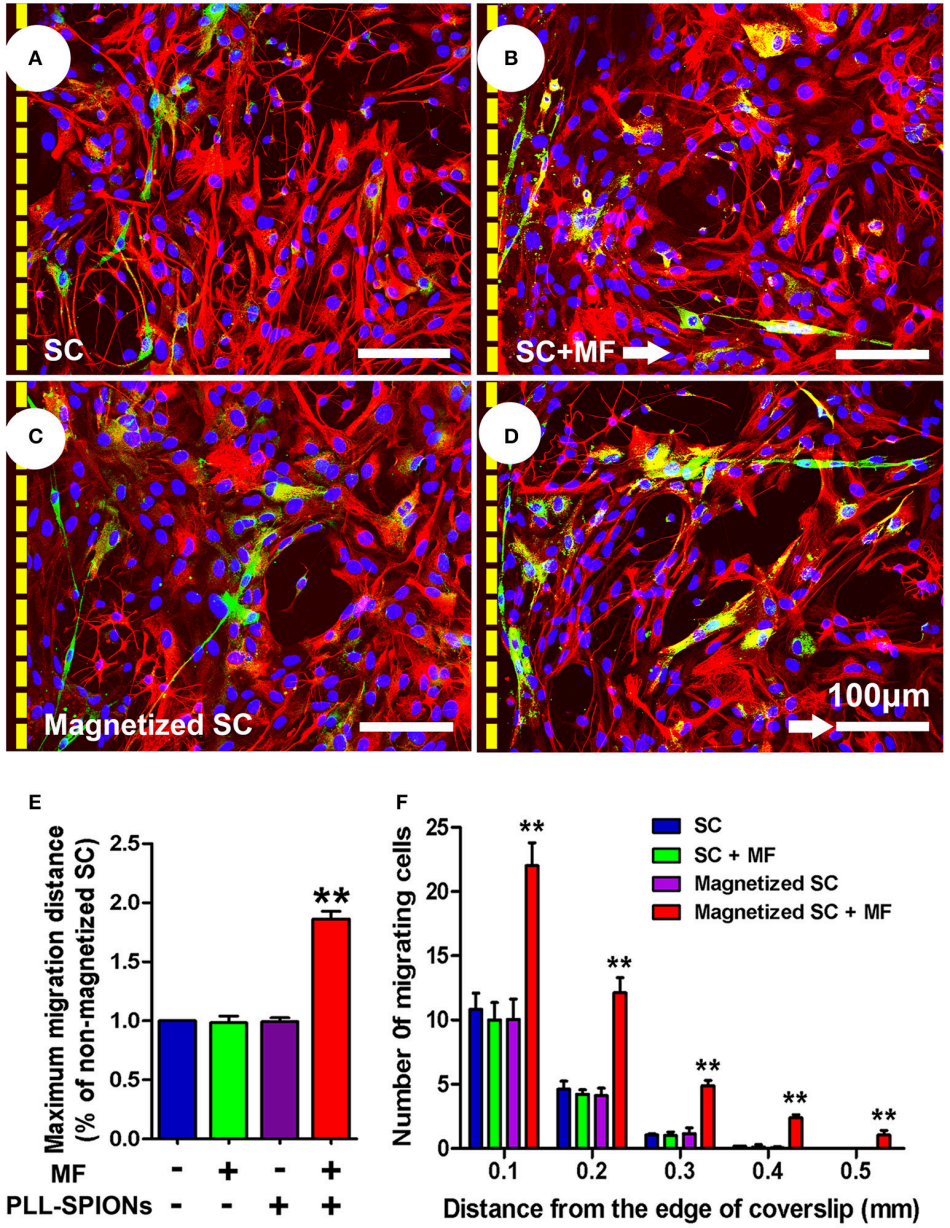
**SC migration assay on astrocytes. (A–D)** SC migration on astrocytes. Astrocytes were only labeled with GFAP (red), while SCs were immunolabeled for P75 (green), nuclei were counterstained with DAPI (blue). Arrows point in the direction of the MF. **(E)** Mean maximum distance traveled from the edge of the inverted fragment. **(F)** Number of cells migrated from the edge of an inverted coverslip. Scale bar = 100 μm. Data are expressed as means ± *SD*; ^**^p < 0.01.

To investigate whether the migration of magnetized SCs on astrocytes could be enhanced under an applied MF, we used an SC migration assay on astrocyte monolayers over a 48 h period (Figure 6). The number of magnetized SCs migrating from the inverted coverslips onto the astrocyte monolayer was increased by 2.55-fold in the presence of a MF compared to that without field stimulation (^**^p < 0.01). No significant differences were found among non-magnetized SCs with or without a MF, and magnetized SCs without a MF (*p* > 0.05). In addition, the maximum migration distance on astrocyte monolayers was significantly longer in magnetized SCs in the presence of a MF than that in the other three groups (^**^p < 0.01; Figure 6E).

### Magnetized SCs no longer form boundaries with astrocytes under an applied MF

**Figure 7:**
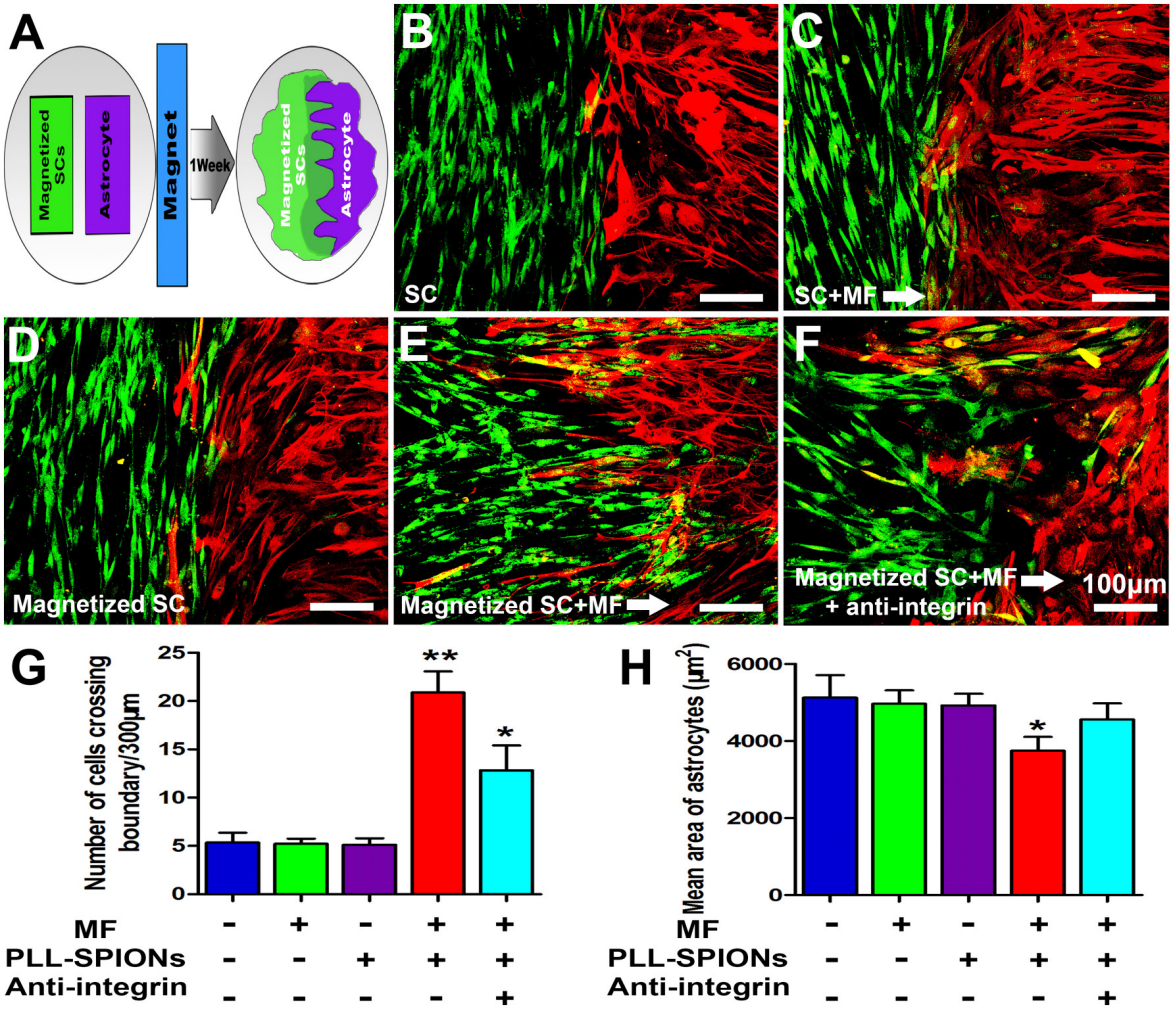
**SC-astrocyte confrontation assay. (A)** A schematic drawing showing that magnetized SCs and astrocytes were seeded in parallel strips and were allowed to migrate and intermingle for 1 week under an applied MF. **(B–F)** Representative images of the SC-astrocyte confrontation assay. **(G)** Quantification of the confrontation assay. SCs were immunolabeled for P75 (green) while astrocytes were immunolabeled for GFAP (red). **(H)** Quantification of mean area of astrocytes in contact with SCs. Arrows point in the direction of the MF. Scale bar = 100 μm. Data are expressed as means ± *SD*; ^**^p < 0.01,^*^p < 0.05.

We next examined whether magnetized SCs could penetrate across the astrocyte boundary and intermingle with the astrocytes under an applied MF (Figure 7A). A clear boundary against astrocytes was observed in non-magnetized SCs with or without a MF, and magnetized SCs without a MF (Figures 7B–D). The number of cells migrated into the sub-region of astrocytes was not significantly different among non-magnetized SCs with or without a MF, and magnetized SCs without a MF (*p* > 0.05). In contrast, the boundary against astrocytes was no longer clear for magnetized SCs in the presence of a MF, and the magnetized SCs intermingled with astrocytes under magnetic stimulation (Figure 7E). Further, analysis showed that the number of cells crossing the astrocyte-SC boundary into the astrocyte domain was 20.89 ± 2.18 cells per 300 μm boundary in magnetized SCs with a MF, which was significantly higher than that in magnetized SCs without a MF; 5.33 ± 1.04 cells per 300 μm boundary (Figure 7G, ^**^p < 0.01). Quantification of the mean areas of GFAP immunoreactivity of astrocytes that in contact with SCs (Figure 7H), it was found that the sizes of astrocytes in contact with magnetized SCs under a MF (3749.74 ± 357.80 μm^2^) were significant smaller than the groups in contacted with non-magnetized SCs with or without a MF, and magnetized SCs without a MF (4820.96 ± 358.34 μm^2^, 4962.06 ± 340.96 μm^2^, and 4920.51 ± 308.48 μm^2^, respectively; ^*^p < 0.05).

### Inhibition of integrin activation caused reduced magnetized SC migration in an astrocyte-rich environment under an applied MF

As shown in Figure 7F, when 10 μg/ml of integrin blocking antibody was added to the culture medium, the number of magnetized SCs migrating into astrocytes under an applied MF was 12.45 ± 2.43 cells per 300 μm boundary, significantly lower than that when the integrin blocking antibody was not applied, but remaining higher than that in the groups with non-magnetized SCs with or without a MF, and magnetized SCs without a MF. Furthermore, application of integrin blocking antibody resulted in significant increase of the cytoplasmic areas of astrocytes compared with magnetized SCs under a MF (4557.42 ± 418.454 μm^2^ vs. 3749.74 ± 357.80 μm^2^; ^*^p < 0.05).

## Discussion

In the present study, SCs were magnetized with PLL-coated SPIONs and PLL-SPIONs at concentrations <20 μg/ml showed negligible toxicity for SCs. In addition, PLL-SPIONs could be efficiently incorporated into SCs, and the amount of incorporated SPIONs was increased as concentration increased. Internalization of SPIONs or the application of a MF alone, had no effect upon the migration ability of SCs. In contrast, magnetized SCs exhibited enhanced migration capacity and migrated along the force direction in the presence of a MF. Interestingly, magnetized SCs showed active migration on astrocytes and intermingled with astrocytes under an applied MF. Furthermore, Inhibition of integrin activation decreased magnetized SCs migration in astrocytes-rich under an applied MF. Thus, SPIONs are efficient agents for the magnetically-driven “actuation” of SC migration. These findings raise the potential to promote the migration of transplanted SCs across the SC-astrocyte interface by magnetic force.

The surface properties of SPIONs are of great importance in terms of their efficient internalization into cells. Due to the negative charge of the cell membrane and SPIONs, naked SPIONs cannot efficiently be internalized into target cells (Nam et al., 2013). PLL is a synthetic amino acid chain with a positive charge and is widely used as a transfection and coating material to enhance cell attachment and adhesion (Wang et al., 2012). In the present study, PLL was used to modify SPIONs via electrostatic interactions. PLL-SPIONs showed positive charges as reported previously (Riggio et al., 2012). In addition, it has been reported that no detectable uptake of iron was found for dextran-coated SPIONs with concentrations <0.5 mg/ml after 48 h incubation, and approximately half of the cells were abundantly labeled at 2 mg/ml (Dunning et al., 2004). In the present study, the internalization of PLL-SPIONs was observed at a concentration as low as 5 μg/ml after just 24 h of incubation, and almost all of the cells were abundantly labeled at 10 μg/ml. These results indicated that our PLL-coated SPIONs are more preferable for efficient SC magnetization.

Although SPIONs are approved by the FDA, concerns have still been raised on their potential for toxic side effects, including impaired mitochondrial function, the formation of apoptotic bodies, the generation of reactive oxygen species (ROS) and DNA damage (Singh et al., 2010). In the present study, we wanted to achieve maximum PLL-SPION internalization but with minimum cytotoxicity. Quantification of intracellular SPIONs showed that the amount of internalized SPIONs increased as the concentration of PLL-SPIONs was increased. However, the cytotoxicity of PLL-SPIONs showed a dose-dependent relationship. We found that PLL-SPIONs with a concentration of 20 μg/ml showed no statistically significant cytotoxicity for SC magnetization. However, cell viability showed a downwards trend. Thus, to avoid potential cytotoxicity and achieve maximum SPION internalization, we chose a concentration of 10 μg/ml as the optimal loading amount for our SPIONs. PCR results showed no differences in the expression of GDNF, NGF, BDNF, and NT-3 between control cells and cells incubated with 10 μg/ml of PLL-SPIONs, indicating that a proper concentration of PLL-SPIONs had no effect upon SC function. Similarly, previous studies have demonstrated that SCs labeled with magnetic particles show normal myelinating ability in spinal cord (Dunning et al., 2004, 2006). Collectively, our findings therefore showed that SCs remained viable and survived under our optimal loading concentration of SPIONs, and that SCs were highly sensitive to the presence of SPIONs at a high dose with a prolonged incubation time. Therefore, it is of great importance to now develop an optimal incubation protocol.

Quantitative analysis showed that that the number of internalized SPIONs reduced overtime. In accordance with this, Franlin et al. demonstrated that the magnetic resonance imaging (MRI) detection of SPION-labeled SCs *in vivo* showed signal loss over time due to cell division (Dunning et al., 2004, 2006). A reduction in the number of internalized SPIONs will reduce the magnetic force acting per cell, which should be taken into consideration for *in vivo* application. On the other hand, a reduced SPION load further indicates that the optimal loading amount of SPIONs has no effect upon cell function, such as proliferation.

The influence of SPIONs upon the migration of different types of cells remains controversial. Human mesenchymal stem cells loaded with SPIONs showed a reduction in migration capacity, with their differentiation potential preserved (Schafer et al., 2009). Another study showed that the internalization of SPIONs partially inhibited the migration of neural stem cells (Cromer et al., 2013). However, it has also been shown that SPION-labeling resulted in enhanced migration in endothelial progenitor cells and human mesenchymal stromal cells (Li et al., 2013; Carenza et al., 2014; Schulze et al., 2014). In the present study, our results demonstrated that SPION-loaded SCs showed no significant change in their migration ability compared to their non-magnetized counterparts. However, in the presence of a MF, magnetized SCs exhibited enhanced cell migration toward the magnetic source. Thus, it is possible that a tensile force drives the magnetized SCs to migrate in a directed orientation.

It is of great importance to increase the number of SCs migrating across the astrocyte boundary and enhance the integration of grafted SCs and host astrocytes, which could promote regenerating axons to depart from the transplants and navigate to their distal destination. It has been reported that the over-expression of PSA-NCAM can enhance SC migration, axonal regrowth and functional recovery following SCI (Papastefanaki et al., 2007; Luo et al., 2011; Ghosh et al., 2012). In addition, various genetic engineering and biochemical approaches have also been introduced to promote SC migration, including providing attractive cues, and the knockdown of aggrecan or N-cadherin (Deng et al., 2011). In the present study, we attempted to increase the number of SCs migrating across the astrocyte boundary and enhance the integration of grafted SCs and host astrocytes via a SPION-mediated tensile force. Under a MF, the boundary against astrocytes was no longer clear for magnetized SCs, and more magnetized SCs penetrated across the cell-cell boundary and into the astrocyte domain. In addition, the enhanced migration of magnetized SCs occurs in a controlled and preferable direction toward the magnetic source. Enhanced directional migration holds the promise to represent a more efficient approach to increase the number of SCs migrating across the astrocyte boundary than enhanced migration with no preferred migrational direction. Furthermore, the controlled and preferable alignment of SCs can induce the formation of bands of Büngner (Ribeiro-Resende et al., 2009), which serve as a “bridge” to guide and foster axons elongating to their distal target organ.

The enhanced integration of SCs and astrocytes might be ascribed to the direct magnetic force which drives SC migration. Recent studies have indicated that cells can also sense the physical force and convert it into biochemical signals which participate in migration, proliferation and differentiation (Sniadecki, 2010; Tseng et al., 2012; Wang, 2014). Integrin has been demonstrated to mediate mechanotransduction. Furthermore, recent studies have demonstrated that CSPGs produced by astrocytes can disrupt the function of integrins in SCs (Afshari et al., 2010). The administration of Mn^2+^ can activate integrin and results in increased SC migration in an astrocyte-rich environment. We therefore investigated whether activated integrin mediates mechanotransduction in the magnetic force driving SC migration into astrocytes. Our results showed that the inactivation of integrin resulted in partial inhibition of the enhanced migratory ability of magnetized SCs in an astrocyte-rich environment under a MF. Thus, the activation of integrin participates in the mechanotransduction underlying the magnetic force driving SC migration into astrocytes. Interestingly, pushing the SCs through the astrocyte by magnetic force didn't result in more reactivity GFAP increase. In contrast, the sizes of astrocytes in contact with magnetized SCs under a MF were significant smaller than astrocytes in contacted with non-magnetized SCs with or without a MF, or magnetized SCs without a MF. Application of integrin blocking antibody resulted in significant increase of the cytoplasmic areas of astrocytes compared with magnetized SCs under a MF. These results indicated that integrin mediated mechanotransduction also involved in reduced hypertrophy of astrocyte in contact with magnetized SCs under a MF. However, mechanotransduction is a complicated process, and other signal pathways might also play a role in this process. This present study provided preliminary data regarding the mechanotransduction mechanism involved in the magnetically-driven enhanced SC migration in an astrocyte-rich environment and reduced astrocytes hypertrophy contacting with SCs in the presence of magnetic force. Further, studies are needed to elucidate the mechanisms up-stream and down-stream of integrin. In addition, it will be interesting to investigate whether other molecules that might mediate mechanotransduction, such as N-cadherin, aggrecan, ephrins, neuregulin, and fibroblast growth factor which involve in interaction between SCs and astrocytes.

The magnetic force acting upon magnetized SCs under an applied MF allows preferred orientation and enhanced migration of SCs to overcome inhibitory factors. A physical explanation of the obtained results is explained below.

**Figure 8:**
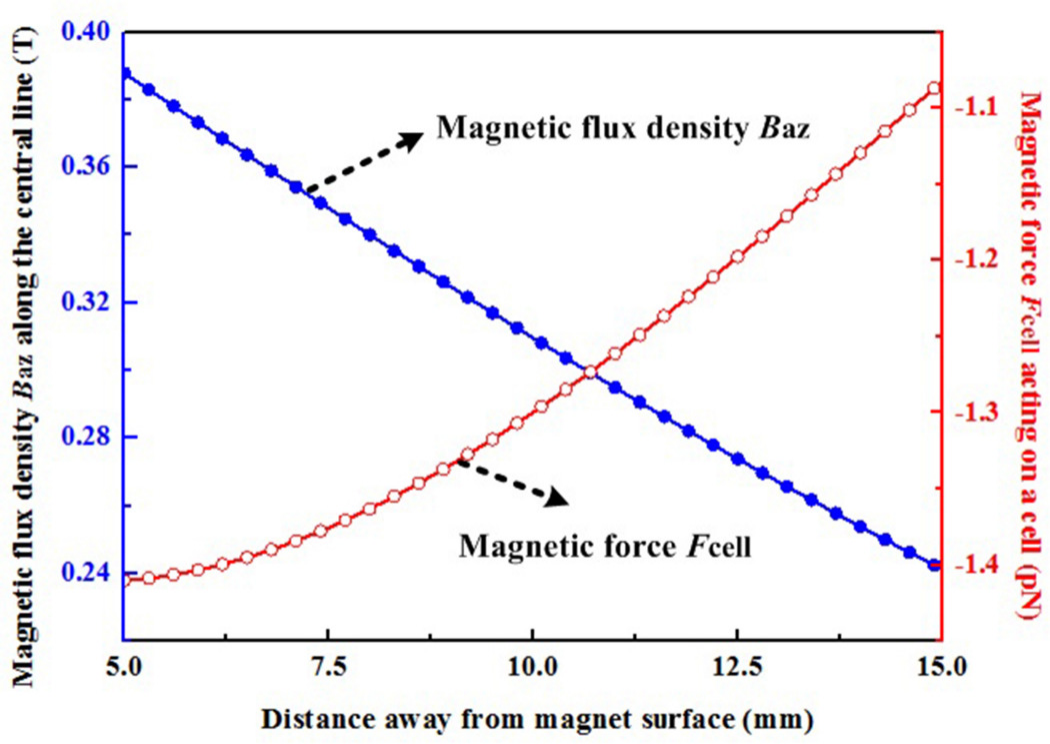
**Magnetic flux density along the z-axis and the magnetic force acting upon a cell**. The negative sign indicates that the directions of these magnetic forces act toward the surface of the permanent magnet.

When magnetic nanoparticles are located in a non-uniform MF, gradient magnetic forces *Fm* acting upon the particles will appear, and can be predicted using an effective dipole moment approach, in which magnetic nanoparticles are modeled as equivalent points with a magnetic dipole moment ***m***. The general expression for these magnetic forces is shown in Equation (1)

(1) $$\begin{array}{r} \text{F}\text{m}=\left(\text{m}▪\nabla \right)\text{B} \end{array}$$

where ***B*** is the magnetic flux density of the applied MF and the magnetic dipole moment ***m*** is given by ***m*** = *V*_*p*_***M***_*p*_. *V*_*p*_ and ***M***_*p*_ are the volume of magnetic nanoparticles with a diameter of 25 nm and the particle magnetization per unit volume, respectively. According to Figure 2C, the strength of ***M***_*p*_ is approximately proportional to the magnitude of the external field in a low field and will tend to reach a value of saturation magnetization ***M***_*ps*_ (351.6 kA/m) when the applied MF is larger than 0.2 T. In our experiment, a rectangular NdFeB permanent magnet (50-mm high, 50-mm long, and 25-mm thick) with a residual magnetic flux density of 1.4 T (B_r_) along the thickness direction was used to generate a non-uniform MF. The three-dimensional magnetic flux density distribution *B* = (*B*_*ax*_, *B*_*ay*_, *B*_*az*_) of this magnet can be obtained by employing an analytical expression (Furlani, 2001), shown in equations (2)–(4).

$$\begin{array}{r} B_{ax}=\frac{M_{r}}{4\pi }\mu_{0}\overset{2}{\underset{i=1}{\sum }}\overset{2}{\underset{j=1}{\sum }}{\left(-1\right)}^{i+j} \end{array}$$

(2) $$\begin{array}{r} \times \ln\;\left\{\frac{\left(y-y_{1}\right)+{\left[{\left(x-x_{i}\right)}^{2}+{\left(y-y_{1}\right)}^{2}+{\left(z-z_{j}\right)}^{2}\right]}^{1/2}}{\left(y-y_{2}\right)+{\left[{\left(x-x_{i}\right)}^{2}+{\left(y-y_{2}\right)}^{2}+{\left(z-z_{j}\right)}^{2}\right]}^{1/2}}\right\} \end{array}$$

$$\begin{array}{r} B_{ay}=\frac{M_{r}}{4\pi }\mu_{0}\overset{2}{\underset{i=1}{\sum }}\overset{2}{\underset{j=1}{\sum }}{\left(-1\right)}^{i+j} \end{array}$$

(3) $$\begin{array}{r} \times \ln\;\left\{\frac{\left(x-x_{1}\right)+{\left[{\left(x-x_{1}\right)}^{2}+{\left(y-y_{i}\right)}^{2}+{\left(z-z_{j}\right)}^{2}\right]}^{1/2}}{\left(x-x_{2}\right)+{\left[{\left(x-x_{2}\right)}^{2}+{\left(y-y_{i}\right)}^{2}+{\left(z-z_{j}\right)}^{2}\right]}^{1/2}}\right\} \end{array}$$

$$\begin{array}{r} B_{az}=\frac{M_{r}}{4\pi }\mu_{0}\overset{2}{\underset{i=1}{\sum }}\overset{2}{\underset{j=1}{\sum }}\overset{2}{\underset{k=1}{\sum }}{\left(-1\right)}^{i+j+k} \end{array}$$

(4) $$\begin{array}{r} \times \tan^{-1}\left\{\frac{\left(x-x_{i}\right)\left(y-y_{j}\right)}{\left(z-z_{k}\right){\left[{\left(x-x_{i}\right)}^{2}+{\left(y-y_{j}\right)}^{2}+{\left(z-z_{k}\right)}^{2}\right]}^{1/2}}\right\} \end{array}$$

where *M*_*r*_ = 1.114 × 10^6^ A/m is the residual magnetization of the permanent magnet, which is obtained by *B*_*r*_/μ_0_ ($\mu_{0}=4\pi \times 10^{-7}\;H/m$). The other symbols (x_1_ = −25 mm, *x*_2_ = 25 mm, *y*_1_ = −25 mm, *y*_2_ = 25 mm, *z*_1_ = −12.5 mm, *z*_2_ = 12.5 mm) in the above equations represent one half of the dimensions of the magnet in the *x, y*, and *z* direction, respectively. Since the magnetic nanoparticles are mainly located along the center line of the permanent magnet in this experiment, the surrounding magnetic field, and the corresponding magnetization of particles, are dominated by the z-axis component. It can be seen that the values of magnetic flux density *B*_*az*_ in the region between 5 and 15 mm away from the permanent magnet, where the magnetic nanoparticles are located, are all larger than 0.2 T (Figure 8). Thus, these particles will be all saturated, and then based on Equation (1), the magnetic force along z-axis *F*_*mz*_ can be calculated by use of equation (5) as follows:

(5) $$\begin{array}{r} F_{mz}=V_{p}M_{r}\frac{dB_{az}}{dz} \end{array}$$

where *V*_*p*_ = 8.18 × 10^−24^ m^3^ for magnetic nanoparticles with a diameter of 25 nm (Table S1), *dB*_*az*_/*dz* means the magnetic field gradient which can be obtained by the first derivative of magnetic field *B*_*az*_ in Equation (3).

The number of internalized SPIONs was calculated from the corresponding internalized iron, and it was found that ~1.27 pg/cell of iron corresponded to ~4.5 × 10^4^ entrapped magnetic particles per cell (***N**p*). A total force ***F**cell* along the z-axis exerted on a single cell was given by equation (4) multiplied by the number ***N***_*p*_: ***F***_*cell*_ = ***N***_*p*_***F***_*mz*_. Thus, the calculated magnetic forces acting upon a cell in the region between 5 and 15 mm away from the permanent magnet range from 1.08 to 1.41 pN. Moreover, based on the above analysis, the force ***F***_*cell*_ can be further increased by increasing the number of entrapped magnetic particles, the volume of magnetic particles, the magnetization of the particles and the MF gradient. The above parameters should be comprehensively optimized by taking account of the preparation of functionalized magnetic nanoparticles, the experimental set-up and the internalization process, and these will be particularly relevant for *in vivo* experiments. Astrocytes play a crucial role in inhibiting SC migration in the CNS environment. Thus, the present study focused upon investigating whether magnetized SCs can be driven into the astrocyte domain and intermingle with astrocytes. Considering the complexity of spinal tissue, a more complex environment, such as slice cultures might be more valuable for future investigations.

In summary, our results validated the hypothesis that SPION-loaded SCs could be driven to migrate across the astrocyte boundary and intermingle with astrocytes in the presence of a MF. Furthermore, integrin-mediated mechanotransduction is involved in the magnetically-driven migration of SCs in an astrocyte-rich environment. Further, work is now needed to validate these findings *in vivo* using animal models of SCI.

## Author contributions

JH and ZJL conceived and designed the experiments. LH wrote the manuscript. BX and ZYL performed the experiments and analyzed the results. QC designed the magnetic field equipment and analyzed the magnetic force.

### Conflict of interest statement

The authors declare that the research was conducted in the absence of any commercial or financial relationships that could be construed as a potential conflict of interest.
