# Supplementary material for: Superparamagnetic Iron Oxide Nanoparticle-Mediated Forces Enhance the Migration of Schwann Cells Across the Astrocyte-Schwann Cell Boundary In vitro
Source: Front Cell Neurosci. 2017 Mar 28;11:83. doi: 10.3389/fncel.2017.00083 (PMC5368970; doi:10.3389/fncel.2017.00083)
Supplement: Supplementary file 1 [file Table1.DOCX]

**Table S1. Related parameters for magnetic force calculation.**

| *Parameter* | Descriptions | Value |
| --- | --- | --- |
| *ρ* | SPIONs density | 4.75×10^3^ kg/m^3^ |
| *V_p_* | Volume of internalized SPIONs | 8.18×10^-24^ m^3^ |
| *N_p_* | Number of internalized SPIONs per cell | ~ 4.5×10^4^ |
