# Supplementary material for: Superparamagnetic Iron Oxide Nanoparticle-Mediated Forces Enhance the Migration of Schwann Cells Across the Astrocyte-Schwann Cell Boundary In vitro
Source: Front Cell Neurosci. 2017 Mar 28;11:83. doi: 10.3389/fncel.2017.00083 (PMC5368970; doi:10.3389/fncel.2017.00083)
Supplement: Supplementary file 2 [file Image1.PDF]

Figure S1

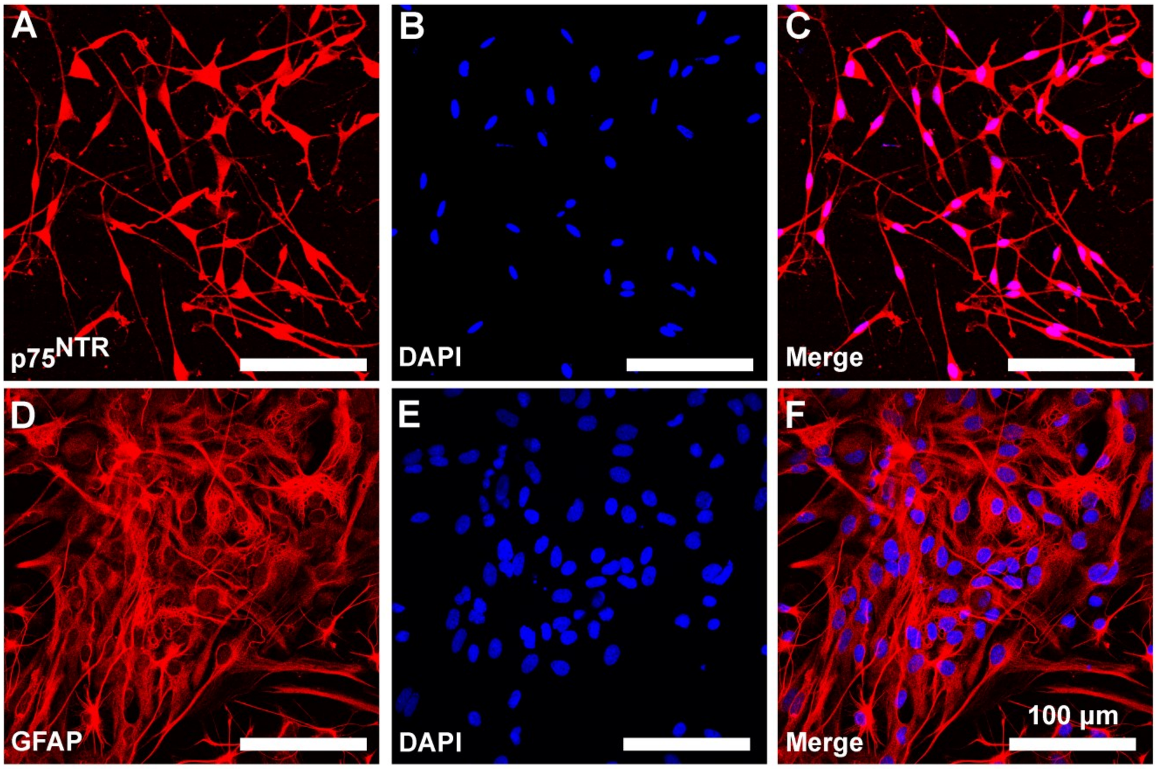

Figure S1: Immunohistochemistry of purified SCs and astrocytes. (A-C) Immunofluorescent staining showed SC specific marker p75NTR was abundantly expressed in the purified cells. The cell nuclei were counterstained with DAPI. Merge files showed a purity of more than 95%. (D-F) Immunofluorescent staining of astrocytes. GFAP was abundantly expressed in the purified astrocytes. The cell nuclei were counterstained with DAPI. Merge files showed a purity of more than 90%. Scale bars = 100 µm.
